# Sin3a drives mesenchymal-to-epithelial transition through cooperating with Tet1 in somatic cell reprogramming

**DOI:** 10.1186/s13287-022-02707-4

**Published:** 2022-01-24

**Authors:** Jiabao Feng, Fugui Zhu, Dan Ye, Qingquan Zhang, Xudong Guo, Changsheng Du, Jiuhong Kang

**Affiliations:** 1 Clinical and Translational Research Center of Shanghai First Maternity and Infant Hospital, Shanghai Key Laboratory of Maternal Fetal Medicine, Shanghai Key Laboratory of Signaling and Disease Research, Frontier Science Center for Stem Cell Research, National Stem Cell Translational Resource Center, School of Life Sciences and Technology, Tongji University, 1239 Siping Road, Shanghai, 200092 People’s Republic of China; 2 Institute for Advanced Study, Tongji University, Shanghai, 200092 People’s Republic of China; 3 Key Laboratory of Spine and Spinal Cord Injury Repair and Regeneration of Ministry of Education, Orthopaedic Department of Tongji Hospital, School of Life Sciences and Technology, Tongji University, 1239 Siping Road, Shanghai, 200092 People’s Republic of China

**Keywords:** Reprogramming, Sin3a, Mesenchymal-to-epithelial transition, Tet1, Hydroxymethylation

## Abstract

**Background:**

Identifying novel regulatory factors and uncovered mechanisms of somatic cell reprogramming will be helpful for basic research and clinical application of induced pluripotent stem cells (iPSCs). Sin3a, a multifunctional transcription regulator, has been proven to be involved in the maintenance of pluripotency in embryonic stem cells (ESCs), but the role of Sin3a in somatic cell reprogramming remains unclear.

**Methods:**

RNA interference of Sin3a during somatic cell reprogramming was realized by short hairpin RNAs. Reprogramming efficiency was evaluated by the number of alkaline phosphatase (AP)-positive colonies and *Oct4*-GFP-positive colonies. RNA sequencing was performed to identify the influenced biological processes after Sin3a knockdown and further confirmed by quantitative RT-PCR (qRT-PCR), western blotting and flow cytometry. The interaction between Sin3a and Tet1 was detected by coimmunoprecipitation. The enrichment of Sin3a and Tet1 at the epithelial gene promoters was measured by chromatin immunoprecipitation. Furthermore, DNA methylation patterns at the gene loci were investigated by hydroxymethylated DNA immunoprecipitation. Finally, Sin3a mutants that disrupt the interaction of Sin3a and Tet1 were also introduced to assess the importance of the Sin3a–Tet1 interaction during the mesenchymal-to-epithelial transition (MET) process.

**Results:**

We found that Sin3a was gradually increased during OSKM-induced reprogramming and that knockdown of Sin3a significantly impaired MET at the early stage of reprogramming and iPSC generation. Mechanistic studies showed that Sin3a recruited Tet1 to facilitate the hydroxymethylation of epithelial gene promoters. Moreover, disrupting the interaction of Sin3a and Tet1 significantly blocked MET and iPSC generation.

**Conclusions:**

Our studies revealed that Sin3a was a novel mediator of MET during early reprogramming, where Sin3a functioned as an epigenetic coactivator, cooperating with Tet1 to activate the epithelial program and promote the initiation of somatic cell reprogramming. These findings highlight the importance of Sin3a in the MET process and deepen our understanding of the epigenetic regulatory network of early reprogramming.

**Supplementary Information:**

The online version contains supplementary material available at 10.1186/s13287-022-02707-4.

## Background

Terminally differentiated cells can be reprogrammed into embryonic stem-like cells, named induced pluripotent stem cells (iPSCs) [1–3]. The development of iPSC technology not only improves our new understanding of cell fate transition but is also helpful for developing iPSC-derived strategies in both basic and clinical studies [4, 5]. Recent studies based on high-throughput sequencing showed that somatic cell reprogramming was driven and dynamically regulated by reprogramming factors (Oct4, Sox2, Klf4, and c-Myc; OSKM), which could bind to the promoter and enhancer regions of different genes during reprogramming and drive iPSC generation [6–10]. However, the underlying mechanisms that control the cell-type transition during reprogramming remain unclear, which raises concern for further clinical applications. Currently, increasing evidence has indicated that mesenchymal-to-epithelial transition (MET) is a rate-limiting step for OSKM-induced fibroblast reprogramming and serves as a prerequisite event for subsequent iPSC maturation and stabilization [11–13]. Blockage of the MET process will result in a significant decrease in reprogramming efficiency. Identifying the key regulators and exact mechanisms driving the MET process would advance our understanding of cell-type transition at the early stage of reprogramming.

Multiple studies have shown that MET, characterized by the silencing of mesenchymal genes and the acquisition of epithelial features, ubiquitously functions in various processes of cell fate transition, including tumorigenesis and embryonic development [14]. During somatic cell reprogramming, reprogramming factors are critical for triggering MET. Oct4, Sox2, and c-Myc inhibit epithelial-mesenchymal transition (EMT) to facilitate MET, while Klf4 directly induces epithelial program [12, 15]. TET family proteins, key molecules for DNA demethylation [16, 17], contribute to pluripotent reprogramming [18, 19]. TET1 acts with NANOG to directly reactivate silenced pluripotency genes, such as *Esrrb* and *Oct4*, and the depletion of Tet1 reduces iPSC generation by mouse embryonic fibroblasts (MEFs) [20]. A related study confirmed that Tet1 could substitute for Oct4 in an OSKM reprogramming cocktail by TET1-mediated hydroxylation of the *Oct4* gene [21]. TET proteins also facilitated the reactivation of the miR-200 family, which is critically important for MET and iPSC generation [15, 22], indicating that TET family proteins are of great significance to the reestablishment of DNA methylation patterns during reprogramming. TET proteins can recognize and bind to CpG dinucleotides through their CXXC zinc finger domain (ZF-CXXC) [23, 24], but it remains unclear how TET proteins regulate the methylation pattern changes in MET and whether other proteins are involved in the precise regulation of DNA methylation remodeling.

Sin3a, as a multifunctional regulator, has been reported to play important roles in cell proliferation, embryogenesis and other processes [25, 26]. Sin3a deficiency often leads to severe DNA damage and growth defects[27]. Mcdonel et al. found that Sin3a was responsible for the genomic stability and pluripotency maintenance of the inner cell mass (ICM) in blastocysts [28]. Our previous study further revealed that knockdown of Sin3a significantly impaired the self-renewal and pluripotency of mouse embryonic stem cells (mESCs) [29], which raised the question of whether Sin3a regulated pluripotency acquisition in somatic cell reprogramming. Previous studies showed that Sin3a could recruit and interact with histone deacetylases (HDACs) by the HID domain to form a corepressive complex, which mediated histone deacetylation and gene repression [30, 31]. However, recent studies reported that the SIN3A/HDAC complex could also play a role in transcriptional activation. For example, the SIN3A/HDAC complex contributes to maintaining pluripotency gene expression in mESCs by cooperating with NANOG [32]. Similarly, Sin3a interacted with Tet1 by its PAH1 domain to activate the transcription of a set of cotarget developmental genes in mESCs [29]. These studies suggested that Sin3a serves as a dual transcriptional regulator, acting as a transcriptional repressor or activator depending on its interacting partners. However, the role of Sin3a in the MET process of early reprogramming and its synergistic partners remain unknown.

In this study, we reported that Sin3a interacted with Tet1, facilitated hydroxymethylation at the promoters of epithelial genes, triggered the MET process and initiated somatic cell reprogramming.

## Methods

### Cell culture

OG-MEFs (*Oct4*-GFP mouse embryonic fibroblasts) were isolated from 13.5-dpc embryos of *Oct4*-GFP transgenic mice [33, 34]. OG-MEFs were cultured in Dulbecco’s modified Eagle medium (DMEM) supplemented with 10% fetal bovine serum (FBS) (Gibco). Early passage (0–3) OG-MEFs were used for somatic cell reprogramming. HEK293FT cells were cultured in DMEM containing 10% FBS. Plat-E cells were cultured in DMEM supplemented with 10% FBS, 1 μg/mL puromycin (Sigma) and 10 μg/mL blasticidin S (Sigma). The iPS cells were cultured on gelatin-coated plates in KOSR medium consisting of knockout DMEM (Gibco), 20% KOSR (Gibco), 100 µM nonessential amino acids (NEAAs) (Gibco), 2 mM L-glutamine (Gibco), 1 mM sodium pyruvate (Gibco), and 55 µM β-mercaptoethanol (Gibco) with leukemia inhibitory factor (LIF) (Millipore).

### Plasmid construction

The vectors used for OG-MEF reprogramming in this study were pMX-Oct4, Sox2, Klf4, and c-Myc [1]. DNA fragments corresponding to the cDNA sequence of Sin3a were amplified by PCR and cloned into the lentivirus vector FUGW. The mutant vectors of Sin3a were obtained by using the QuickChange Lightning Site Directed Mutagenesis Kit (Agilent Technologies catalog no. 210515-5). The shRNAs targeting Sin3a or Tet1 were cloned into the lentivirus vector pLKO.1. All the constructed plasmids were verified by DNA sequencing. The primers used for vector construction are listed in Additional file 1: Table S1.

### Virus preparation and MEF reprogramming

Recombinant plasmids were introduced into HEK293FT cells together with the packaging plasmids Pax2 and Vsvg and the pMX-Oct4, Sox2, Klf4, and c-Myc vectors were transfected into Plat-E cells by Fugene HD transfection reagent (Roche). At 48 h after transfection, the virus-containing supernatant was harvested and filtered through 0.45 μm Millex-HV (Millipore) filters to remove cell debris.

OG-MEFs were seeded at a density of 8 × 10^4^ cells per well in a 12-well plate and incubated overnight with virus-containing supernatant combined with 4 μg/ml polybrene (Sigma). Then, the virus-containing supernatant was replaced with fresh DMEM supplemented with 10% FBS. At 72 h post-infection, the medium was replaced with KOSR medium and changed every two days until GFP^+^ colonies appeared.

For human fibroblast reprogramming, human skin fibroblasts (1 × 10^6^ cells) were transduced with 5 μg nonintegrated episomal iPSC reprogramming vectors (1.25 μg each of pCLXE-hOct3/4-shp53, pCLXE-hSox2-Klf4, pCLXE-hLmyc-Lin28, and pCLXE-GFP) [35] and seeded at a density of 5 × 10^4^ cells per well in a 6-well plate. Three days later, fibroblasts were infected with shRNA viruses combined with 8 μg/ml polybrene (Sigma) for 12 h. After one week of induction, the hiPSC derivation medium (DMEM/F12 (Gibco) supplemented with 20% KOSR, 100 µM nonessential amino acids (NEAA), 1 mM GlutaMAX, 55 µM β-mercaptoethanol, and 10 ng/mL bFGF (R&D)) was changed every two days.

### Quantitative RT–PCR analysis

Total RNA was extracted from cells by using RNAiso Plus (TaKaRa), and cDNA synthesis was performed by using a PrimeScript RT reagent kit (TaKaRa). qRT-PCR analysis was performed using SYBR Green qPCR Master Mix (Bio-Rad) on an Mx3000P qPCR System (Agilent). The expression levels of genes of interest were normalized to glyceraldehyde-3-phosphate-dehydrogenase (GAPDH), and the relative expression level was calculated by the 2^−ΔΔCt^ method. The primers used for qRT-PCR are listed in Additional file 1: Table S2.

### Western blotting

Cells were harvested and lysed with SDS lysis buffer supplemented with a 1× Protease Inhibitor Cocktail (Roche). Protein lysates were separated by SDS**–**PAGE, blotted onto polyvinylidene difluoride (PVDF) membranes, blocked in 3% BSA-TBST for 1 h and incubated with primary antibodies overnight at 4 °C. Membranes were washed and incubated with appropriate secondary antibodies for 1 h at room temperature. Signals were visualized by enhanced chemiluminescence (ECL) (ImageQuant LAS 4000 mini). The primary antibodies used in western blotting were anti-Sin3a (Abcam, ab3479), anti-E-cadherin (Abcam, ab11512), anti-β-catenin (Abcam, ab16051), and anti-GAPDH (Bioworld, AP0063). GAPDH was used as the loading control.

### Alkaline phosphatase (AP) staining

After fixation with 4% paraformaldehyde (PFA) and washing with 1× phosphate-buffered saline (PBS), AP staining was performed by using alkaline phosphatase kits (Sigma) according to the manufacturer’s protocol.

### Chromatin immunoprecipitation (ChIP)

ChIP assays were carried out as previously described [36]. The antibodies used in ChIP were anti-Sin3a (Abcam, ab3479), anti-Tet1 (Genetex, GTX124207), anti-HDAC1 (Santa Cruz Biotechnology, SC-8410), and rabbit IgG (Millipore). Immunoprecipitated DNA and input DNA were used as templates for qRT-PCR analysis. The primers used for ChIP-qPCR are listed in Additional file 1: Table S3.

### Coimmunoprecipitation

The reprogrammed cells were harvested and lysed with cell lysis buffer (1% Triton X-100 in 50 mM Tris–HCl, pH 7.4 containing 150 mM NaCl, 2 mM Na_3_VO_4_, 100 mM NaF, and protease inhibitors). 10% of the cell lysate was used for Input. The rest of the lysate was incubated with antibody overnight at 4 °C and the target protein was captured by a 1:1 mixture of Ezview Red Protein A Affinity Gel (Sigma, P6486) and Ezview Red Protein GAffinity Gel (Sigma, E3403) for 4 h at 4 °C. The immunoprecipitated proteins were used to perform western blotting. The antibodies used in coimmunoprecipitation were anti-Sin3a (Abcam, ab3479), anti-Tet1 (Genetex, GTX124207), anti-HDAC1 (Santa Cruz Biotechnology, SC-8410), anti-Flag (CST, 14793s), and rabbit IgG (Millipore). Rabbit IgG was used as a negative control.

### Hydroxymethylated DNA Immunoprecipitation (hMeDIP)

Genome DNA was extracted by a genomic DNA extraction kit (TIANGEN). Then, fragmentation of purified genomic DNA was performed by using the restriction enzyme Bfa1 overnight at 37 °C. After fragmentation, single-stranded DNA was obtained by incubating for 10 min at 99 °C. And ten percent of the total volume of the genomic DNA fragments was kept as input. The rest of the DNA fragments were immunoprecipitated with the antibody 5hmC (Active Motif, 39769) for 3 h at 4 °C. After incubation, the DNA-antibody complexes were captured by ChIP-grade protein G magnetic beads (CST) for 3 h at 4 °C. The immunoprecipitated DNA was purified by using MinElute PCR purification kits (Qiagen) and further quantified by qRT-PCR. Fold enrichment was calculated relative to input DNA. The primers used for hMeDIP-qPCR are listed in Additional file 1: Table S4.

### Flow cytometry

First, the reprogramming cells were dissociated by 0.25% trypsin/EDTA, washed with PBS and blocked in PBS containing 1% BSA for 1 h at room temperature. Then, the cells were stained with primary antibody (diluted in PBS containing 1% BSA) for 30 min at room temperature. After washing, the cells were incubated with Alexa Fluor-488- or Alexa Fluor-594-conjugated secondary antibodies (Invitrogen) for 30 min at room temperature. Finally, the cells were analyzed with BD FACSVerse and FlowJo software. The antibodies used in flow cytometry were anti-E-cadherin (Abcam, ab11512), anti-β-catenin (Abcam, ab16051), donkey anti-rat IgG (H + L) Alexa Fluor 488 (Life Technologies, A21208), and donkey anti-rabbit IgG (H + L) Alexa Fluor 594 (Life Technologies, A21207).

### RNA sequencing and data analysis

Total RNA was extracted from the reprogramming cells by RNAiso Plus (TaKaRa). The RNA-seq was performed by BGISEQ-500 platform. After filtering, the clean reads were mapped to the mouse genome (mm10) from the UCSC Genome Browser database by using Bowtie2 and the expression levels of genes or transcripts were calculated by RSEM. Genes or transcripts with a fold-change in expression > twofold and a *p* value < 0.001 were considered differentially expressed genes (DEGs). DAVID bioinformatic resources were used for Gene Ontology (GO) analysis.

### Statistical analyses

Student's t test (two-tailed) was used for statistical significance analysis in this study. The error bars represent the standard deviation (SD) of three independent biological replicates. */#/$, **/##/$$, and ***/###/$$$ represented *P* < 0.05, *P* < 0.01, and *P* < 0.001, respectively.

## Results

### Sin3a is required for reprogramming from MEFs to iPSCs

**Fig. 1:**
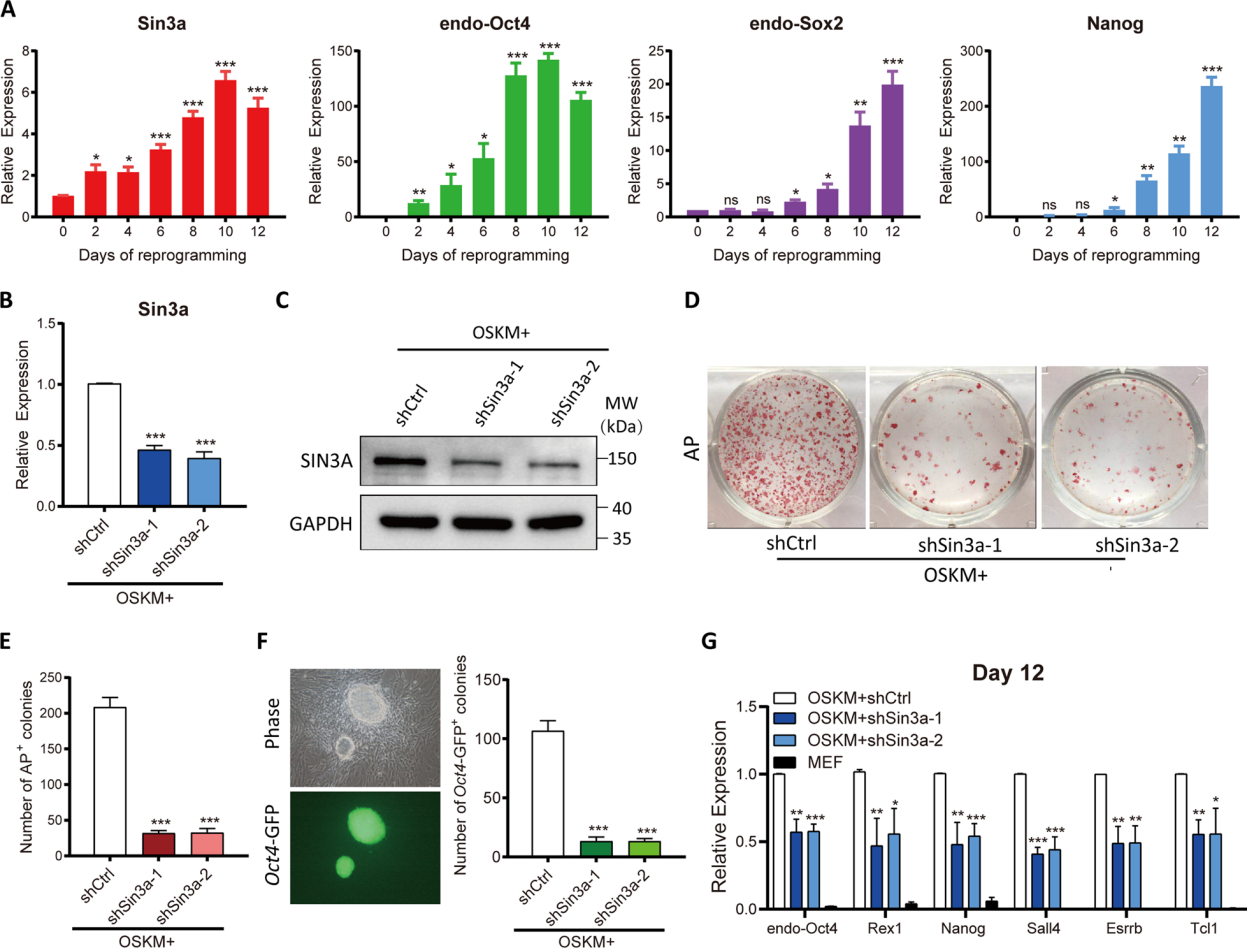
Sin3a is required for somatic cell reprogramming. **A** The expression patterns of Sin3a, endo-Oct4, endo-Sox2, and Nanog at the indicated days (0, 2, 4, 6, 8, 10, and 12) of OSKM-driven MEF reprogramming. **B** The expression of Sin3a in reprogrammed MEFs infected with scramble (shCtrl) or shSin3a (shSin3a-1 and shSin3a-2) viruses at Day 3 of reprogramming. **C** Western blotting analysis of SIN3A expression at Day 3 of reprogramming after Sin3a knockdown. GAPDH was used as loading control. **D**–**E** Representative AP staining (red) images are shown (**D**) and the number of AP^+^ colonies was counted (**E**) at Day 12 of MEF reprogramming. **F** The morphology of GFP^+^ iPSC colonies is shown (left). The number of GFP^+^ colonies was counted at Day 12 (right). **G** qRT-PCR analysis of pluripotency markers (endo-Oct4, Rex1, Nanog, Sall4, Esrrb, and Tcl1) at Day 12 of MEF reprogramming. Data in **A**–**G** presented for three independent experiments. Significance was estimated by student’s unpaired *t* test. *P < 0.05, **P < 0.01, and ***P < 0.001 versus the control group. Error bars represent SD. Abbreviations: shCtrl, control shRNA; OSKM, Oct4, Sox2, Klf4, and c-Myc; AP, alkaline phosphatase; MEF, mouse embryonic fibroblasts; ns, no significance

To determine whether Sin3a regulated somatic cell reprogramming, we surveyed the expression pattern of Sin3a by qRT-PCR during reprogramming. The expression of Sin3a gradually increased in parallel with MEF reprogramming, which occurred earlier than the expression of endogenous pluripotency markers (Oct4, Sox2, and Nanog) (Fig. 1A), suggesting that Sin3a might be involved in the initiation of reprogramming. Then, we designed two independent short hairpin RNAs (shSin3a-1 and shSin3a-2) that specifically targeted the Sin3a transcripts, which was verified via qRT-PCR and western blotting (~ 40–50% knockdown efficiency) (Fig. 1B, C). The reprogramming efficiency was measured by AP staining at Day 12 of reprogramming. We observed that Sin3a inhibition significantly resulted in fewer AP^+^ colonies than the control (Fig. 1D, E). We then introduced shSin3a viruses into OG-MEFs that contained a GFP reporter driven by the *Oct4* promoter and found that knockdown of Sin3a significantly decreased the number of *Oct4*-GFP^+^ colonies on Day 12 of reprogramming (Fig. 1F). We further detected pluripotency gene expression by qRT-PCR and our results showed that there was a remarkable downregulation of pluripotency gene expression after Sin3a inhibition (Fig. 1G). In contrast, overexpression of Sin3a increased the expression of pluripotency genes (Additional file 1: Fig. S1A and S1B). Taken together, these results suggested that Sin3a is necessary for MEF reprogramming.

### Sin3a functions at the early stage of MEF reprogramming

**Fig. 2:**
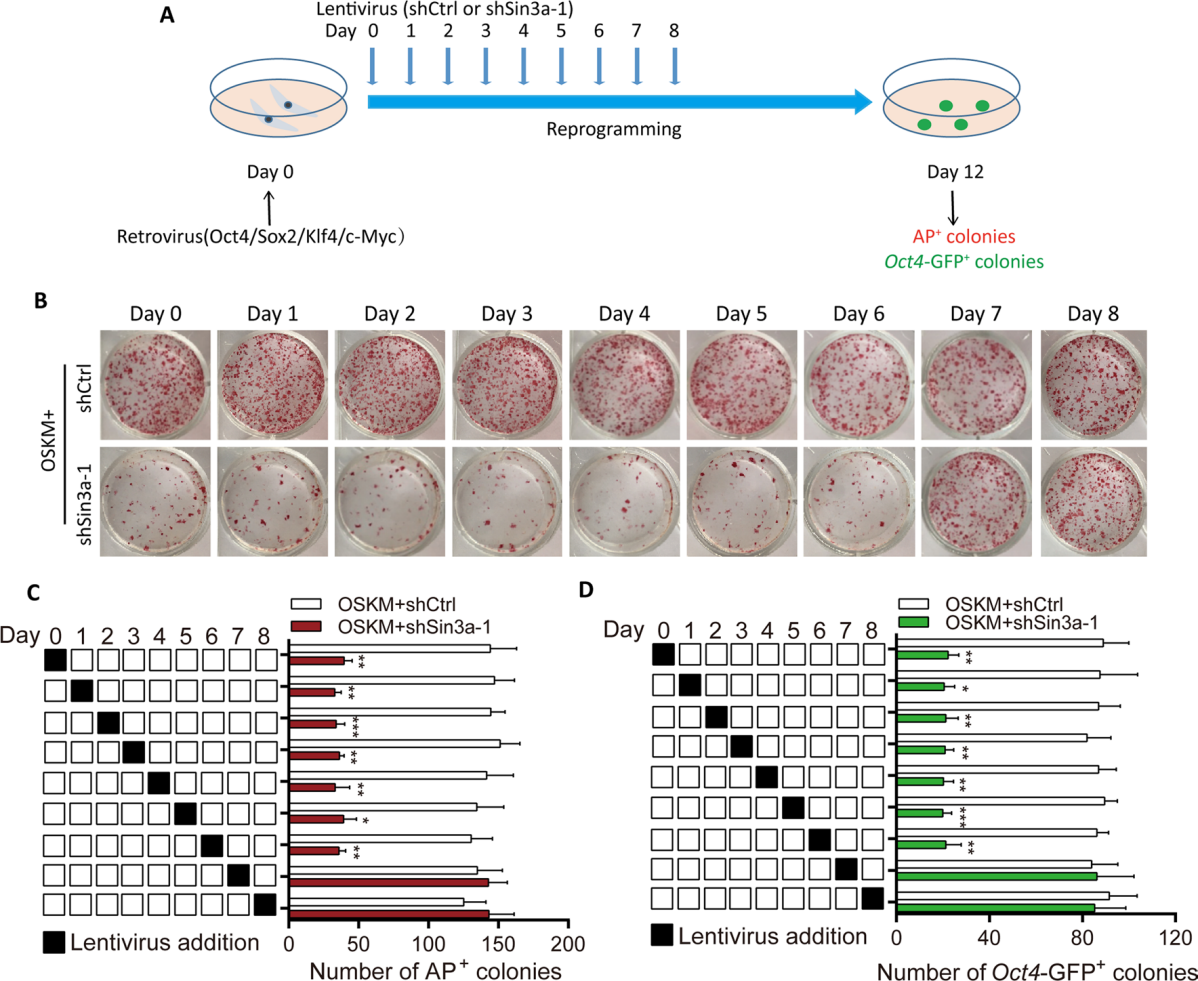
Sin3a contributes to the initiation process of reprogramming. **A** A schematic illustration of the experimental strategy is presented. MEFs were reprogrammed with OSKM retroviruses and infected with a control lentivirus (shCtrl) or a shRNA lentivirus (shSin3a-1) for one day from Day 0 to Day 8. Reprogramming efficiency was measured by counting the number of AP^+^ colonies or GFP^+^ colonies at Day 12. **B** Representative images of AP staining at Day 12 of reprogramming are shown. **C** and **D** The number of AP^+^ colonies (**C**) or GFP^+^ colonies (**D**) generated from MEFs infected with control and shSin3a-1 viruses was counted at Day 12. Significance was estimated by student’s unpaired t test. *P < 0.05, **P < 0.01, and ***P < 0.001 versus the control group. Error bars represent SD (n = 3 independent experiments)

To further investigate the time courses of Sin3a function in MEF reprogramming, we introduced shRNA lentivirus (shSin3a-1) or control lentivirus (shCtrl) into MEFs on the indicated days (Day 0 to Day 8) of reprogramming. Reprogramming efficiency was evaluated by the number of AP^+^ colonies and *Oct4*-GFP^+^ colonies at Day 12 (Fig. 2A). Our findings showed that knockdown of Sin3a led to fewer AP^+^ and *Oct4*-GFP^+^ colonies at the early stage of reprogramming (Day 0 to Day 6), but there was no significant effect on reprogramming efficiency with shSin3a lentivirus addition after Day 6 of reprogramming (Fig. 2B–D). These results indicated that Sin3a mainly functions in the initiation phase of MEF reprogramming.

### Sin3a mainly mediates the MET process of MEF reprogramming

**Fig. 3:**
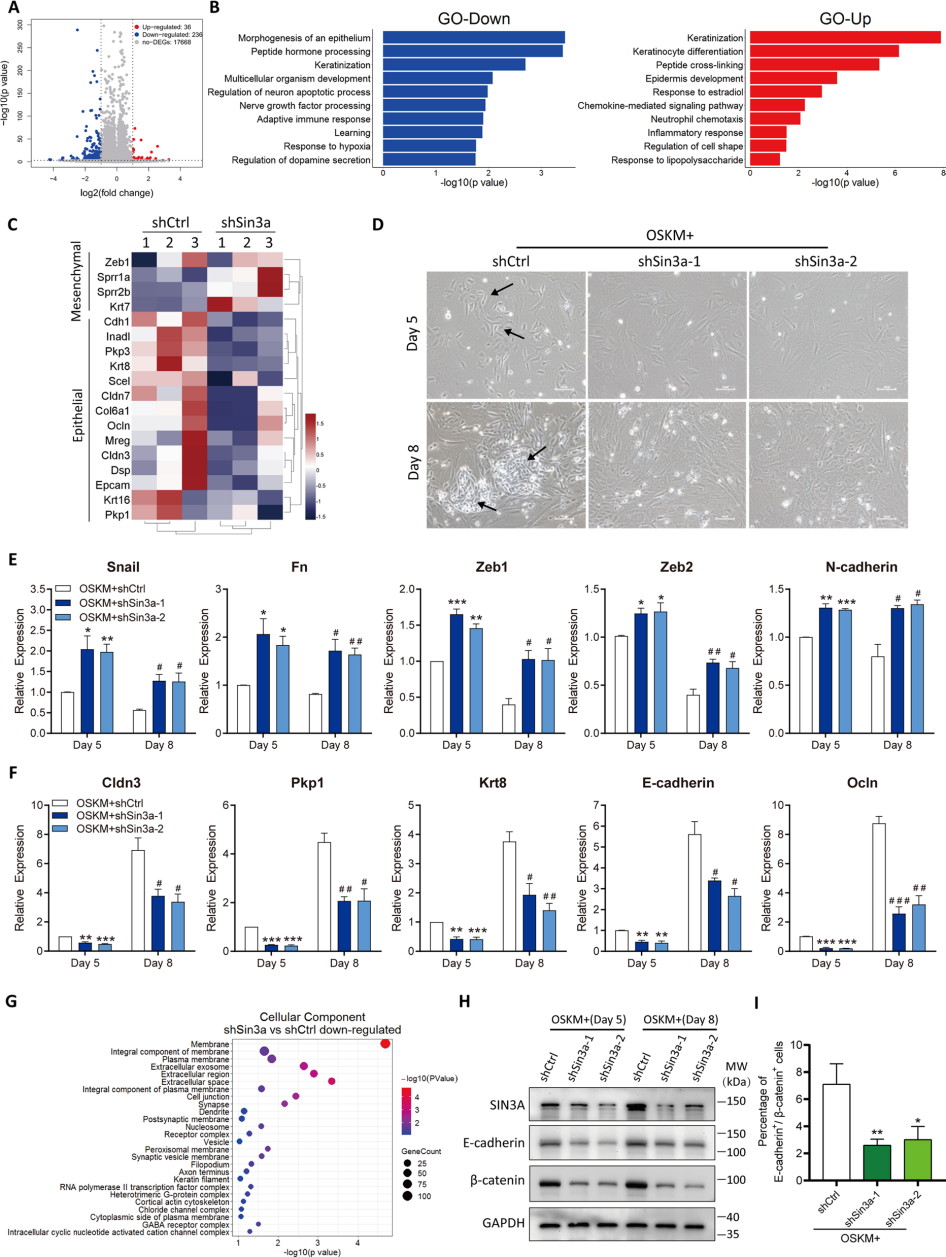
RNA-seq reveals Sin3a deficiency impedes MET process. **A** Volcano plot of differentially expressed genes (red, upregulated genes; blue, downregulated genes) after Sin3a knockdown. **B** The top 10 enriched GO terms for the differentially expressed genes (GO-Down, downregulated genes, left; GO-Up, upregulated genes, right) in the Sin3a knockdown group are shown. **C** Heatmap for the MET-related genes in the shSin3a-1 group compared to the control. **D** Representative images of the cell morphology of the control and Sin3a knockdown groups at Day 5 and Day 8 of MEF reprogramming. Black arrows indicate the representative cell–cell contact morphology. **E**, **F** qRT-PCR analysis of the expression changes of mesenchymal markers (**E**) and epithelial markers (**F**) at Day 5 and Day 8 of MEF reprogramming after Sin3a knockdown. **G** GO term analysis for the significantly downregulated genes in the Sin3a knockdown group is shown. **H** Western blotting analysis of SIN3A, E-cadherin, and β-catenin expression at Day 5 and Day 8 of MEF reprogramming. GAPDH was used as loading control. **I** Flow cytometry detection of the percentage of E-cadherin and β-catenin positive cells in the control and Sin3a knockdown groups at Day 8 of MEF reprogramming. Significance was estimated by student’s unpaired *t* test. Data in **E**, **F**, and **I** were representative of three independent experiments. *P < 0.05, **P < 0.01, and ***P < 0.001 versus the control group at Day 5. ^#^*P* < 0.05, ^##^*P* < 0.01, and ^###^*P* < 0.001 versus the control group at Day 8. Error bars represent SD

To explore the biological processes in which Sin3a was primarily involved, RNA sequencing (RNA-seq) was employed on Day 3 of reprogramming between the control and shSin3a groups (*n* = 3 independent experiments for each group). A total of 17,940 genes were detected in total RNA and we identified 272 genes that were differentially expressed in the shSin3a group compared with the control group (fold-change > 2; *p* value < 0.001). Among these DEGs, we found that 36 genes were significantly upregulated after Sin3a knockdown, whereas 236 genes were significantly downregulated (Fig. 3A). Gene ontology (GO) analysis of the DEGs revealed that the downregulated genes in shSin3a group were related to epithelial cell morphogenesis, whereas the upregulated genes were related to processes specific for fibroblasts, such as keratinization and keratinocyte differentiation (Fig. 3B). We focused on the MET process during reprogramming initiation, and our findings showed that Sin3a knockdown resulted in the increased expression of mesenchymal markers and significantly decreased the expression of epithelial markers (Fig. 3C), suggesting that Sin3a might contribute to the epithelial program and regulate the MET process at the early stage of MEF reprogramming.

To further verify whether Sin3a participated in the regulation of the MET process, cell morphology and marker gene expression were analyzed on Days 5 and 8 of reprogramming. We observed remarkable cell morphological changes, such as cell shrinkage, in the control group on Days 5 and 8 of reprogramming, while knockdown of Sin3a resulted in a dramatic defect in epithelial morphogenesis (Fig. 3D and Additional file 1: Fig. S2). Cell morphological changes during MET were accompanied by upregulation of epithelial genes and downregulation of mesenchymal genes. Our results also showed that Sin3a inhibition significantly hindered the activation of epithelial genes (Cldn3, Pkp1, Krt8, etc*.*) and led to high expression of mesenchymal genes (Snail, Fn, Zeb1, etc.) (Fig. 3E, F). Force expression of Sin3a promoted the morphogenesis of epithelial cells (Additional file 1: Fig. S3A and S3B), along with the inhibition of mesenchymal genes and activation of epithelial genes (Additional file 1: Fig. S3C and S3D). Moreover, we introduced shSIN3A lentivirus into human skin fibroblasts together with reprogramming factors (Additional file 1: Fig. S4A). Our results similarly showed that SIN3A inhibition led to impaired epithelial morphogenesis and downregulation of epithelial gene expression in human somatic cell reprogramming (Additional file 1: Fig. S4B and S4C). We further analyzed the cellular components of downregulated genes and found that knockdown of Sin3a significantly affected the components of the cell membrane, which mediated the establishment of cell–cell adhesion of epithelial cells (Fig. 3G). The cell adhesion molecule E-cadherin and its cytosolic partner β-catenin were involved in the reestablishment of epithelial features. Our results showed that knockdown of Sin3a inhibited the establishment of cell–cell contact by suppressing the expression of E-cadherin and β-catenin (Fig. 3H, I). Taken together, these results strongly suggested that Sin3a plays an important role in the regulation of the MET process and that a lack of Sin3a impedes the initiation of MEF reprogramming.

### Sin3a interacts with Tet1 to regulate the hydroxymethylation of epithelial genes

**Fig. 4:**
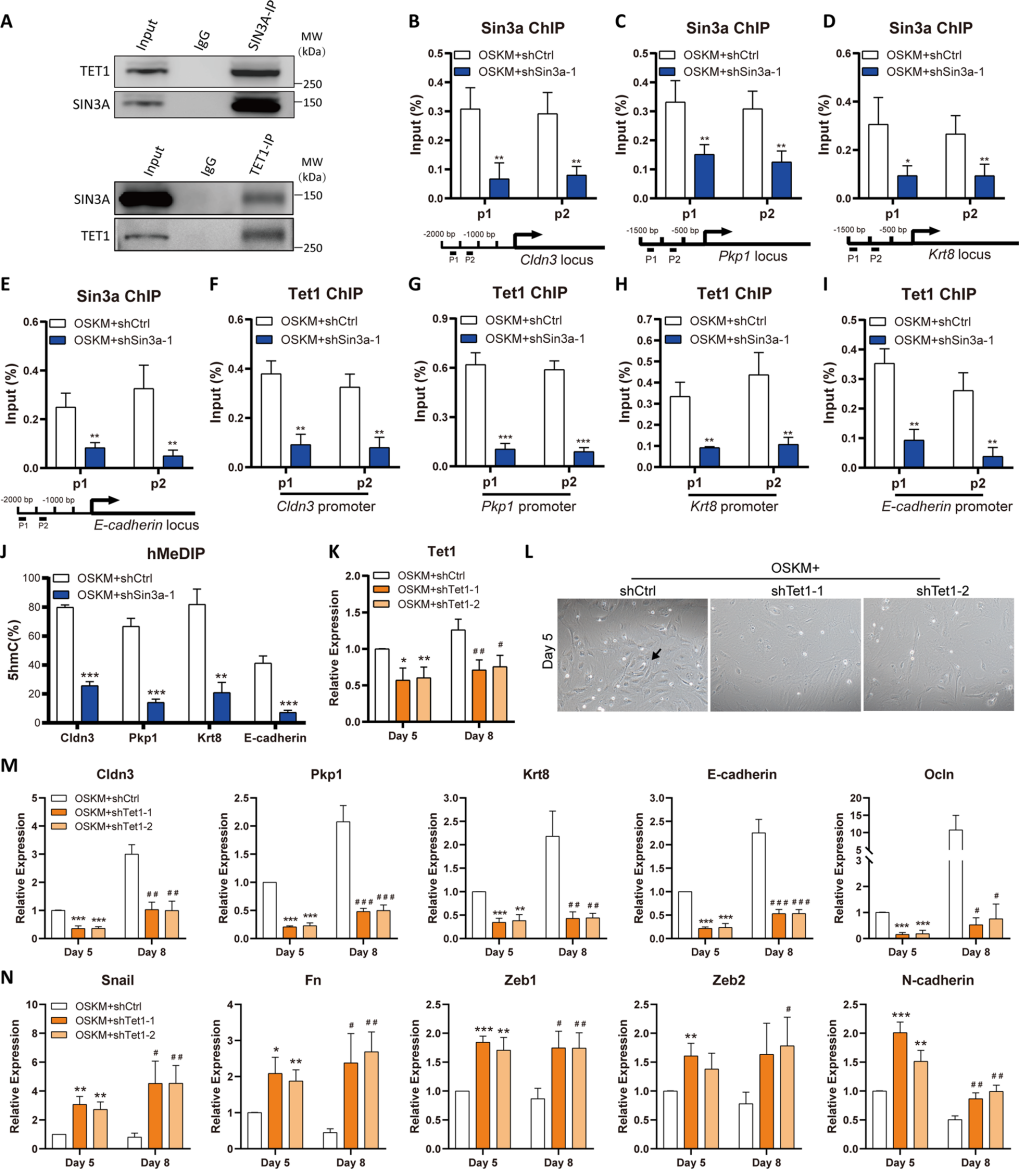
Sin3a recruits Tet1 and facilitates the hydroxymethylation of epithelial gene promoters. **A** Co-IP analysis for the interaction of endogenous SIN3A and TET1 at Day 5 of reprogramming. 10% of total lysates were used for input. Rabbit IgG antibody as the negative control. **B**–**E** ChIP-qPCR analysis (*n* = 4) of Sin3a enrichment at the promoter of epithelial markers (*Cldn3* (**B**), *Pkp1* (**C**), *Krt8* (**D**), and *E-cadherin* (**E**)) at Day 5. The samples were normalized to input DNA. **F**–**I** ChIP-qPCR analysis of Tet1 occupation at the promoter of epithelial genes at Day 5. **J** hMeDIP-qPCR was employed to detect the 5hmC level at the promoter of epithelial markers after Sin3a knockdown. *P < 0.05, **P < 0.01, and ***P < 0.001 versus the control group. **K** qRT-PCR analysis of Tet1 expression at Day 5 and Day 8 of reprogramming. MEFs were infected with scramble (shCtrl) or shSin3a (shTet1-1 or shTet1-2) lentivirus. **L** Representative images of cell morphology at Day 5 of reprogramming. The black arrow indicates the representative epithelial morphogenesis. **M**, **N** qRT-PCR analysis of the expression changes of epithelial genes (**M**) and mesenchymal genes (**N**) at Day 5 and Day 8 of reprogramming after Tet1 knockdown. Significance was estimated by student’s unpaired t test (n = 3 independent experiments). *P < 0.05, **P < 0.01, and ***P < 0.001 versus the control group at Day 5. ^#^*P* < 0.05, ^##^*P* < 0.01, and ^###^*P* < 0.001 versus the control group at Day 8. Error bars represent SD

Our previous study revealed that Sin3a was involved in maintaining the pluripotency of embryonic stem cells by interacting with Tet1 [29]. Thus, we hypothesized that Sin3a might cooperate with Tet1 to regulate the MET process of reprogramming. To verify this hypothesis, the reprogramming cells at Day 5 were harvested and analyzed by coimmunoprecipitation (Co-IP) followed by western blotting. Our results demonstrated that Sin3a endogenously coimmunoprecipitated with Tet1, which was further substantiated by reverse Co-IP (Fig. 4A). We further examined whether Sin3a and Tet1 directly regulated the epithelial gene expression. The Sin3a and Tet1 occupancies were analyzed by chromatin immunoprecipitation followed by qRT-PCR (ChIP-qPCR) at the promoter of epithelial genes. Our results demonstrated that Sin3a directly bound to the promoters of epithelial genes such as *Cldn3, Pkp1, Krt8,* and *E-cadherin,* while the enrichment of Sin3a was significantly reduced after Sin3a knockdown (Fig. 4B–E and Additional file 1: Fig. S5A), suggesting that Sin3a directly regulated the expression of these epithelial genes. More importantly, the expression level of Tet1 was not influenced by Sin3a knockdown, but the enrichment of Tet1 was significantly reduced at the promoter region of epithelial genes after Sin3a knockdown (Fig. 4F–I and Additional file 1: Fig. S5B). On the other hand, overexpression of Sin3a significantly increased the enrichment of Tet1 at the promoter of epithelial genes (Additional file 1: Fig. S6A–D). Consistently, knockdown of SIN3A also led to impaired TET1 occupation at the promoter of epithelial genes during human skin fibroblast reprogramming (Additional file 1: Fig. S7A–C). These results implied that Sin3a may assist Tet1 in binding at the promoter of epithelial genes and directly regulate the expression of these genes.

It has been proven that TET family proteins are responsible for DNA demethylation by generating 5-hydroxymethylcytosine (5hmC). To further provide insight into the regulatory mechanisms at the promoters of epithelial genes, we extracted genomic DNA from reprogrammed cells on Day 5 and examined the 5hmC contents at the promoter regions of epithelial genes. The hMeDIP-qPCR results showed that DNA hydroxymethylation at the promoter regions of epithelial genes was significantly decreased after Sin3a knockdown (Fig. 4J). We further found that knockdown of Tet1 led to impaired epithelial morphogenesis (Fig. 4K, L), inhibition of epithelial genes (Fig. 4M), and upregulation of mesenchymal gene expression (Fig. 4N) during reprogramming, which indicated that Tet1 also played an important role in MET.

In addition, Sin3a is well known for interacting with HDACs to regulate transcription. We also performed Co-IP analysis and showed that Sin3a endogenously interacted with HDAC1 during reprogramming (Additional file 1: Fig. S8A). The enrichment analysis of HDAC1 at the promoter of epithelial genes and mesenchymal genes showed that HDAC1 was only enriched at the promoter region of mesenchymal genes, which was not affected by Sin3a knockdown (Additional file 1: Fig. S8B–I), indicating that the enrichment of HDAC1 at the promoter of mesenchymal genes was independent of Sin3a. Taken together, our data implied that Sin3a regulates the hydroxymethylation and activation of epithelial genes by interacting with Tet1 during the MET process.

### Sin3a-Tet1 interaction is necessary for Tet1 occupation and hydroxymethylation at epithelial gene promoters

**Fig. 5:**
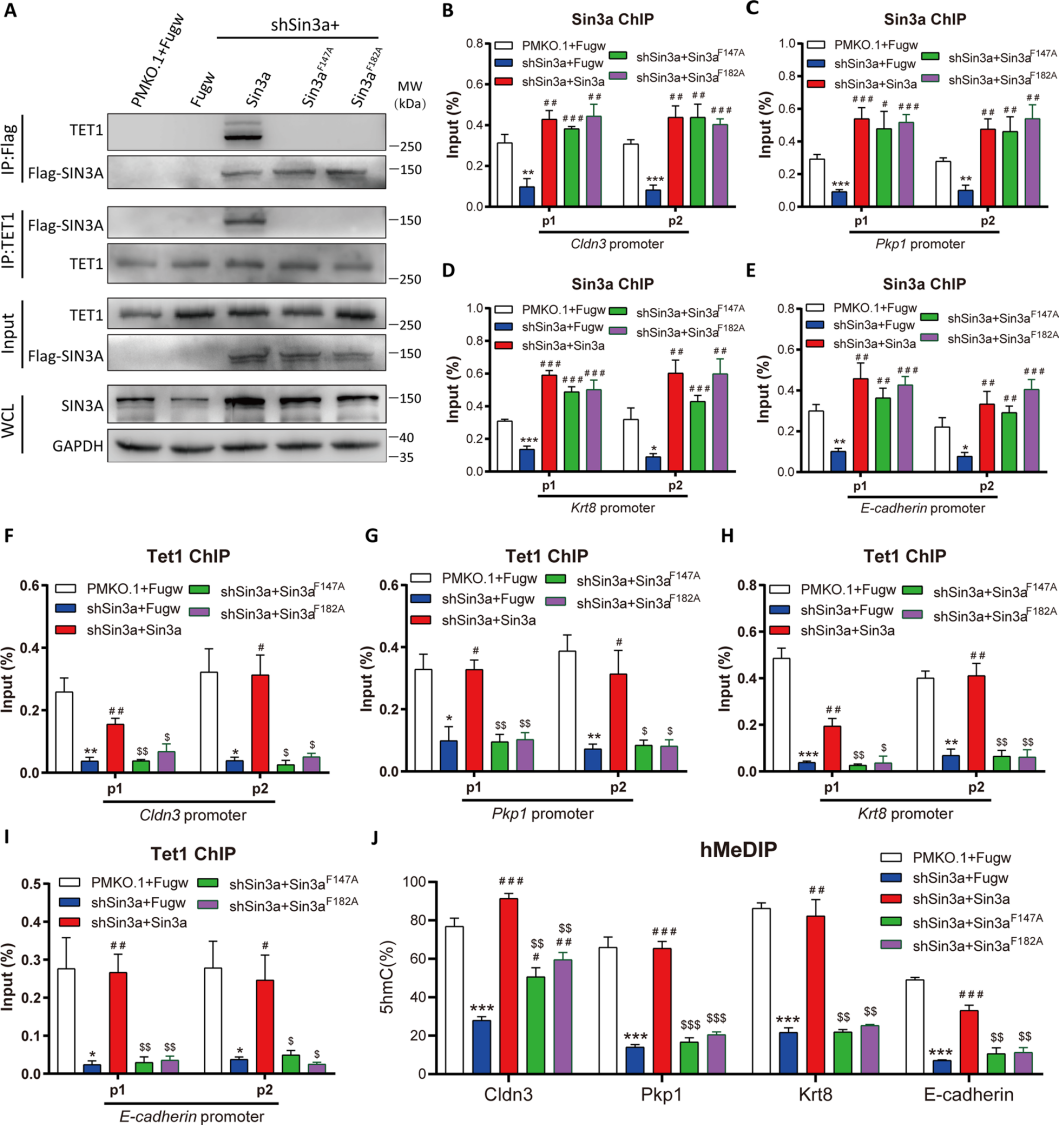
The interaction of Sin3a and Tet1 is critical for Tet1 occupation and hydroxymethylation at the promoter of epithelial genes. **A** Co-IP analysis of the interaction of SIN3A and TET1 at Day 5 in shSin3a-1 cells overexpressing wild-type Sin3a or Sin3a mutants (Sin3a^F147A^ and Sin3a^F182A^). Nonsense mutations were introduced into the wild-type Sin3a and Sin3a mutants to protect against shSin3a-1 targeting. **B**–**E** ChIP-qPCR results (*n* = 4 independent experiments) of Sin3a occupation at the promoter of epithelial markers (*Cldn3* (**B**), *Pkp1* (**C**), *Krt8* (**D**), and *E-cadherin* (**E**)) at Day 5 of reprogramming. **F**–**I** ChIP-qPCR results of Tet1 occupation at the promoter of epithelial markers at Day 5 of reprogramming. **J** hMeDIP-qPCR results of 5hmC levels at the promoter of epithelial markers in shSin3a-1 cells overexpressing wild-type Sin3a or Sin3a mutants. Data in **F**–**J** were representative of three independent experiments. Significance was estimated by student’s unpaired *t* test. Error bars represent SD. *P < 0.05, **P < 0.01, and ***P < 0.001 versus pMKO.1-treated group with FUGW vector (the white column), ^#^*P* < 0.05, ^##^*P* < 0.01, and ^###^*P* < 0.001 versus shSin3a-1-treated group with FUGW vector (the blue column), ^$^*P* < 0.05, ^$$^*P* < 0.01, and ^$$$^*P* < 0.001 versus the wild-type Sin3a overexpression group (the red column)

Our previous research identified two key amino acids (F147A and F182A) in the paired amphipathic helix 1 (PAH1) domain of Sin3a that are responsible for Sin3a-Tet1 binding [29]. To gain insight into the function of Sin3a-Tet1 interaction, we performed rescue experiments using wild-type Sin3a and these two Sin3a mutants (Sin3a^F147A^ and Sin3a^F182A^). First, we confirmed that overexpression of these two Sin3a mutants in shSin3a cells recovered the total expression of Sin3a, which was similar to the effect of wild-type Sin3a overexpression (Additional file 1: Fig. S9A and S9B). Overexpression of wild-type Sin3a or two Sin3a mutants had no influence on Tet1 expression (Additional file 1:Fig. S9C). Then, we performed endogenous Co-IP in reprogramming cells on Day 5 and found that the Tet1 protein indeed interacted with wild-type Sin3a but not the Sin3a mutants (Fig. 5A). We also found that overexpression of both wild-type Sin3a and mutants could increase the enrichment of Sin3a at the promoter of epithelial markers (Fig. 5B–E). However, only wild-type Sin3a rescued the enrichment of Tet1 at the same loci, whereas mutation of two binding sites nearly abolished the enrichment of Tet1 at the promoter of epithelial genes (Fig. 5F–I). Consistently, the DNA hydroxymethylation level at the promoter of these epithelial genes could be completely or partially recovered by wild-type Sin3a but not the mutants (Fig. 5J). Collectively, the results demonstrated that the binding of Tet1 and Sin3a is crucial for Tet1 occupation and hydroxymethylation at epithelial gene promoters.

### Sin3a-Tet1 interaction is required for the MET process and iPSC generation

**Fig. 6:**
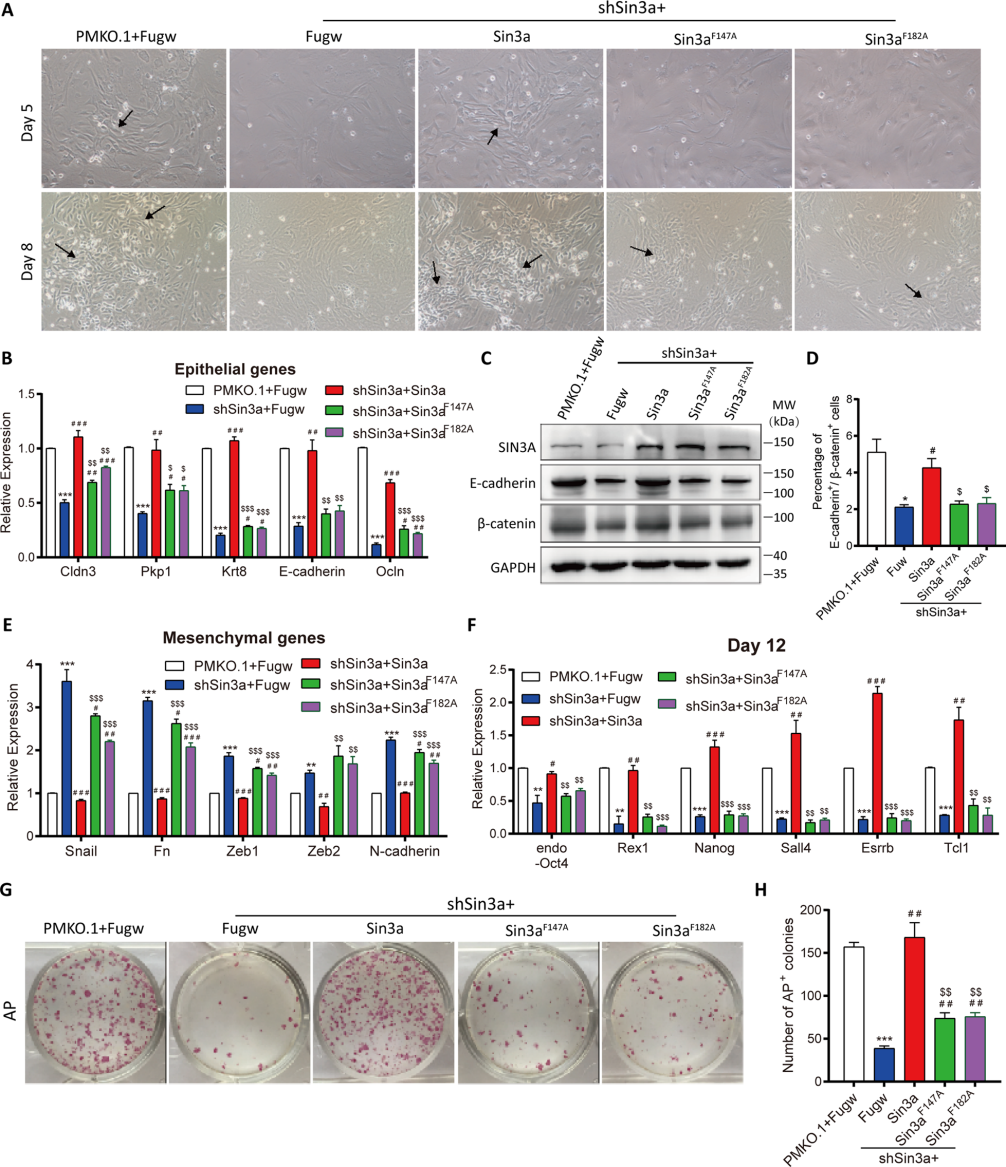
Sin3a cooperates with Tet1 to promote MET and somatic cell reprogramming. **A** The cell morphology of wild-type Sin3a or Sin3a mutants (Sin3a^F147A^ and Sin3a^F182A^) overexpression in shSin3a-1 cells at Day 5 and Day 8 of MEF reprogramming. Black arrows indicate the representative cell–cell contact morphology. **B** qRT-PCR analysis of the expression changes of epithelial genes at Day 8 in shSin3a-1 cells overexpressing wild-type Sin3a or Sin3a mutants. **C** Western blotting analysis of SIN3A, E-cadherin, and β-catenin expression at Day 8 of MEF reprogramming. GAPDH was used as loading control. **D** Flow cytometry detection of the percentage of E-cadherin and β-catenin positive cells at Day 8 of reprogramming. **E** qRT-PCR analysis for the expression changes of mesenchymal genes at Day 8 of reprogramming. **F** qRT-PCR analysis of pluripotency marker expression at Day 12 of MEF reprogramming. **G**–**H** Reprogramming efficiency was measured by AP staining. Representative images (**G**) and the number of AP^+^ colonies (**H**) are shown. Significance was estimated by student’s unpaired t test. Error bars represent SD (*n* = 3 independent experiments). *P < 0.05, **P < 0.01, and ***P < 0.001 versus pMKO.1-treated group with fugw vector (the white column), ^#^*P* < 0.05, ^##^*P* < 0.01, and ^###^*P* < 0.001 versus shSin3a-1-treated group with fugw vector (the blue column), ^$^*P* < 0.05, ^$$^*P* < 0.01, and ^$$$^*P* < 0.001 versus the wild-type Sin3a overexpression group (the red column)

To determine whether the binding of Sin3a and Tet1 was necessary for MEF reprogramming, cell morphological changes were further observed with light microscopy on Days 5 and 8. The morphological changes induced by knocking down Sin3a could be recovered by wild-type Sin3a but not the mutants (Fig. 6A). Consistently, overexpression of wild-type Sin3a rescued the impaired epithelial features caused by Sin3a inhibition, including the downregulation of epithelial genes and suppression of E-cadherin and β-catenin, but the mutants failed to do so (Fig. 6B–D and Additional file 1: Fig. S10A). Furthermore, the upregulation of mesenchymal markers induced by Sin3a knockdown could be completely restored by wild-type Sin3a but not Sin3a mutants (Fig. 6E and Additional file 1: Fig. S10B). This evidence shows that the Sin3a-Tet1 interaction plays an essential role in the MET process.

Next, we determined the reprogramming efficiency by qRT-PCR and AP staining on Day 12. The impaired reprogramming caused by Sin3a knockdown was fully recovered by overexpressing wild-type Sin3a, whereas disrupting the interaction of Sin3a and Tet1 failed to restore either the pluripotency gene expression or the number of AP^+^ colonies (Fig. 6F–H). Taken together, our findings concluded that the Sin3a-Tet1 interaction is necessary for the effective reprogramming of MEFs to iPSCs.

## Discussion

Multiple studies have reported that Sin3a is not only involved in the regulation of the cerebral cortex and lung development [37, 38] but also plays a pivotal role in biological processes, including cell survival, proliferation, and aging [26, 27]. A recent study showed that SIN3A colocalized with NANOG at the promoter of pluripotency genes to maintain the pluripotency of mESCs [32]. Our previous study also reported that Sin3a directly activated the expression of Lefty1, an antagonist of the Nodal signaling pathway, thereby contributing to pluripotency maintenance mESCs [29]. This evidence indicated that Sin3a was a pivotal regulator in the maintenance of pluripotency in mESCs. However, the role of Sin3a in pluripotency acquisition during reprogramming remains poorly understood. Our results showed that the expression of Sin3a gradually increased during MEF reprogramming, and knockdown of Sin3a at the beginning of reprogramming significantly reduced the reprogramming efficiency, suggesting that Sin3a contributes as a new functional regulator in the initiation phase of somatic cell reprogramming.

The MET process is considered as the critical rate-limiting event of MEF reprogramming, which has a great impact on the efficiency of reprogramming. Previous studies have indicated that various factors, such as signaling pathways, noncoding RNAs, and transcription factors, constitute the complex regulatory network of the MET processes [39–41]. For example, systematic RNAi screening has revealed that BMP signaling is crucial for inducing the initiation of the MET process at the early stage of MEF reprogramming [13]. The miR-200 family was also reported to facilitate epithelial morphogenesis and drive MET. In addition, YTHDF2 and YTHDF3, m^6^A modification recognizers, were found to promote MET and MEF reprogramming by mediating the degradation of Tead2 mRNA [42]. It has been reported that SIN3A cooperates with NANOG to improve the established pluripotency during late-stage reprogramming [30]. However, the roles of Sin3a at the initiation stage of MEF reprogramming, especially MET, remain poorly understood. In this study, our results showed that silencing Sin3a expression at early days significantly blocked efficient reprogramming. We further demonstrated that Sin3a deficiency impaired the reprogramming efficiency and MET progress by hindering the activation of the epithelial program in MEF reprogramming and human skin fibroblast reprogramming, while forced expression of Sin3a significantly facilitated the MET process. For the first time, we revealed that Sin3a is a new upstream facilitator of the MET process in the initiation phase of cell reprogramming.

Previous studies have shown that ectopic expression of Tet proteins could reactivate the silenced pluripotency gene and promote iPSC generation [43–45]. For example, Tet1/2 cooperate with NANOG to establish pluripotency during reprogramming [20]. Tet2 is crucial for the formation of 5-hydroxymethylcytosine (5hmC) at pluripotency gene loci, whereas knockdown of Tet2 could significantly block the reactivation of pluripotency genes such as *Nanog* and *Esrrb*, which leads to reduced reprogramming efficiency [18]. Notably, Tet1 was also reported to facilitate DNA demethylation and reactivation of the *Oct4* gene during reprogramming, and Tet1 could even replace Oct4 to generate fully pluripotent iPSCs conjoining with Sox2, Klf4, and c-Myc [21]. In addition, the TET family indirectly promoted the MET process and iPSC generation by inducing the oxidative demethylation and reactivation of *miR-200s* [22]. In this study, we verified that Tet1 deficiency hindered epithelial feature acquisition at the early stage of reprogramming. More importantly, with the assistance of Sin3a, Tet1 could directly bind to the promoter of epithelial genes and activate their transcription, thereby triggering MET. Therefore, our study provides a new understanding of the epigenetic regulatory role of the TET enzyme and Sin3a in MET and the initiation of somatic cell reprogramming.

Sin3a contains multiple protein-interaction domains that are responsible for interacting with partners and executing distinct roles in various biological processes. Previous studies have mainly focused on the transcriptional repressive roles of Sin3a by forming the SIN3A/HDAC corepressor complex. For example, the SIN3A/HDAC complex interacted with the transcription repressor FOXN3 in estrogen receptor-positive (ER^+^) cells, mediating the silencing of epithelial genes and promoting EMT [46]. The SIN3A/HDAC complex was also reported to suppress the cotranscriptional accumulation of harmful R-loops and prevent DNA damage by cooperating with the conserved RNA-binding factor THO [47]. Surprisingly, recent studies found that Sin3a could also play essential roles in transcriptional activation. SIN3A/HDAC physically interacted with NANOG to activate pluripotency gene expression and promote pluripotency [32]. Our previous study also revealed that Sin3a was responsible for the recruitment of Tet1 to the promoter region of the *Lefty1* gene and activating the transcription of a series of downstream genes [29]. This evidence suggested that the unique roles of Sin3a depended on its interacting partners. Chandru et al. identified that the conserved Sin3 interaction domain (SID) of Tet1 was indispensable for the interaction between Sin3a and Tet1 [48]. In this research, we found that mutations in the PAH1 domain of Sin3a, disrupting the interaction of Sin3a and Tet1, significantly impaired the activation of epithelial genes, the MET process, and iPSC generation. In addition, we found that Sin3a interacted with both Tet1 and HDAC1 during that MET process. Only Tet1, not HDAC1, could directly bind to the promoter of epithelial genes and trigger MET with the assistance of Sin3a, suggesting that the Sin3a/Tet1 interaction was important and unique for epithelial gene reactivation. Hence, the Sin3a-Tet1 interaction reshaped the DNA methylation landscape of epithelial gene promoters, which was indispensable for triggering the MET process and initiating somatic cell reprogramming.

## Conclusions

**Fig. 7:**
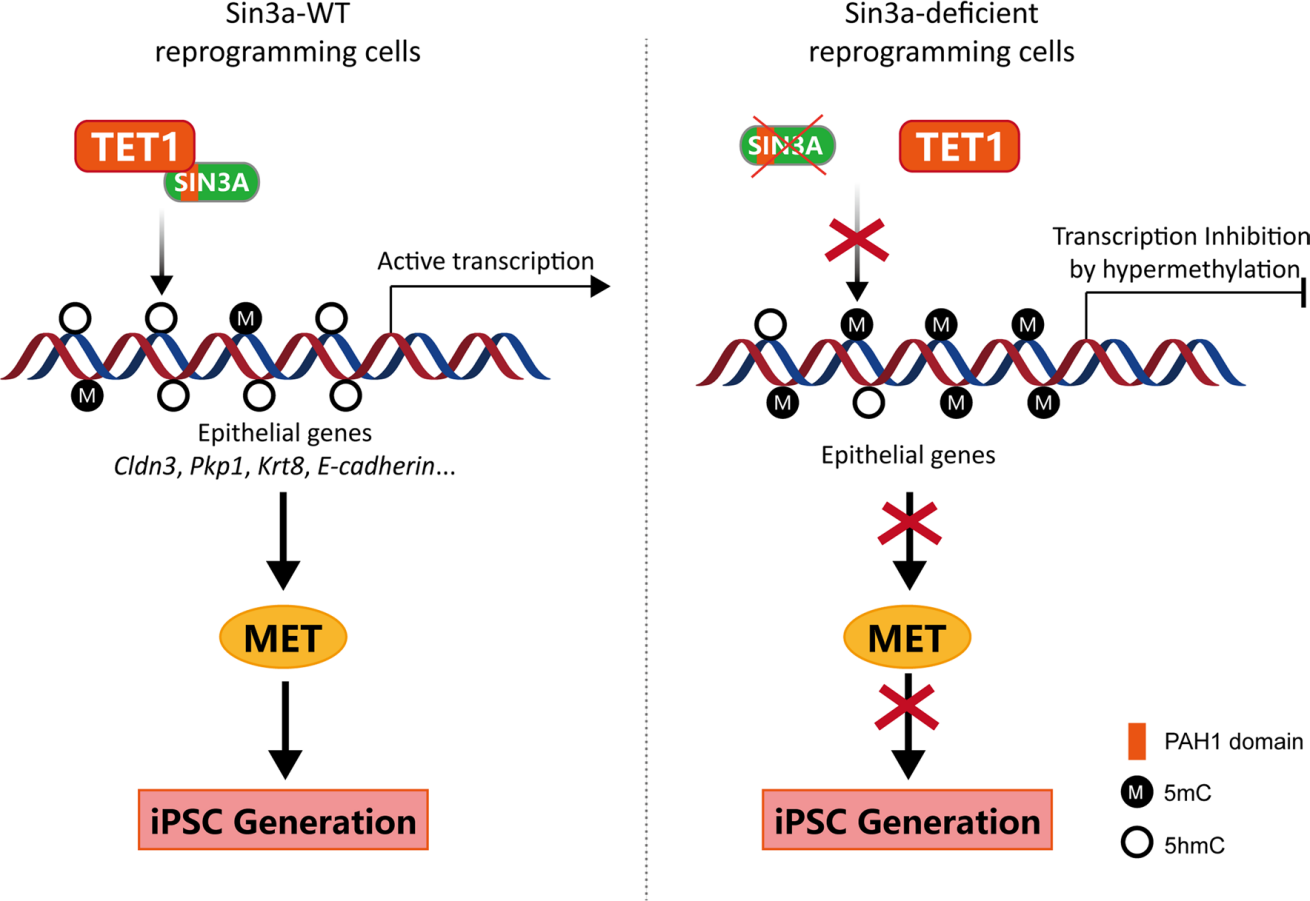
Schematic model for Sin3a mediating MET and somatic cell reprogramming through functional cooperation with Tet1. During somatic cell reprogramming, Sin3a is responsible for guiding Tet1 to the promoter regions of epithelial genes and activating their transcription, thereby triggering MET and initiating somatic cell reprogramming (left). Sin3a deficiency impedes the activation of epithelial gene expression, MET process, and iPSC generation (right)

In summary, we identified that Sin3a was a functional regulator of the initiation of somatic cell reprogramming, which contributed to facilitating the MET process by mechanically cooperating with Tet1 to promote the demethylation and transcriptional activation of epithelial genes (Fig. 7). Our work provides a new insight into DNA methylation remodeling and epigenetic regulation during the initiation of somatic cell reprogramming, especially the MET process.

## Supplementary Information

**Additional file 1**. Supplemental Figures S1–S10 and Tables S1–S4.

## Data Availability

The datasets used and analyzed during the current study are available from the corresponding author on reasonable request.

## Abbreviations

iPSCs: Induced pluripotent stem cells
ESCs: Embryonic stem cells
MEF: Mouse embryonic fibroblast
MET: Mesenchymal-to-epithelial transition
EMT: Epithelial-mesenchymal transition
ZF-CXXC: CXXC zinc finger domain
ICM: Inner cell mass
HDACs: Histone deacetylases
HID domain: Histone interaction domain
PAH1 domain: Paired amphipathic helix 1 domain
GFP: Green fluorescent protein
DMEM: Dulbecco's modified Eagle medium
FBS: Fetal bovine serum
NEAAs: Nonessential amino acids
LIF: Leukemia inhibitory factor
GAPDH: Glyceraldehyde-3-phosphate-dehydrogenase
PVDF: Polyvinylidene difluoride membranes
ECL: Enhanced chemiluminescence
PFA: Paraformaldehyde
ChIP: Chromatin immunoprecipitation
Co-IP: Coimmunoprecipitation
hMeDIP: Hydroxymethylated DNA immunoprecipitation
EDTA: Ethylene diamine tetraacetic acid
PBS: Phosphate-buffered saline
BSA: Bovine serum albumin
DEGs: Differentially expressed genes
GO analysis: Gene ontology analysis
SD: Standard deviation
qRT-PCR: Quantitative real-time polymerase chain reaction
AP: Alkaline phosphatase
SID: Sin3 interaction domain
