# Supplementary material for: Sin3a drives mesenchymal-to-epithelial transition through cooperating with Tet1 in somatic cell reprogramming
Source: Stem Cell Res Ther. 2022 Jan 24;13:29. doi: 10.1186/s13287-022-02707-4 (PMC8785580; doi:10.1186/s13287-022-02707-4)
Supplement: Supplementary file 1 — Additional file 1. Supplemental Figures S1–S10 and Tables S1–S4. [file 13287_2022_2707_MOESM1_ESM.pdf]

Additional file

Supplementary Figures

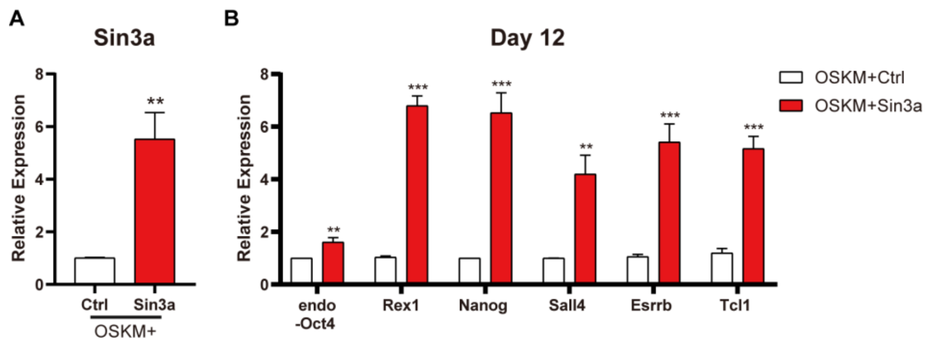

Figure S1. The effect of Sin3a overexpression on pluripotency gene expression during reprogramming. Related to Figure 1.

(A, B) qRT–PCR analysis of the expression of Sin3a (A) and pluripotency genes (B) at Day 12 of reprogramming. Significance was estimated by student's unpaired t-test. (n=3). **P < 0.01 and ***P < 0.001 versus the OSKM+Ctrl group. Error bars represent SD.

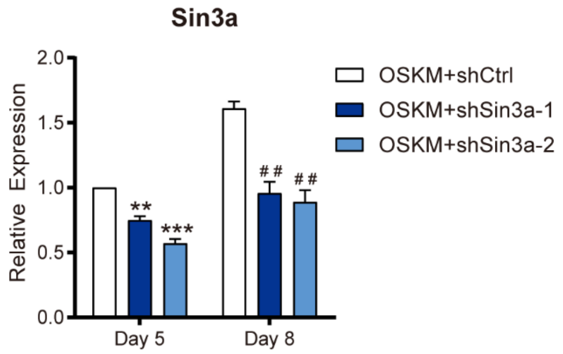

Figure S2. Expression analysis for Sin3a knockdown during MEF reprogramming. Related to Figure 3.

qRT–PCR analysis of Sin3a expression at Days 5 and 8 of reprogramming. MEFs were infected with scramble (shCtrl) or shSin3a (shSin3a-1 or shSin3a-2) viruses.

Significance was estimated by student's unpaired t-test (n=3). **P < 0.01 and ***P < 0.001 versus the control group at Day 5. $^{##}$P < 0.01 versus the control group at Day 8. Error bars represent SD.

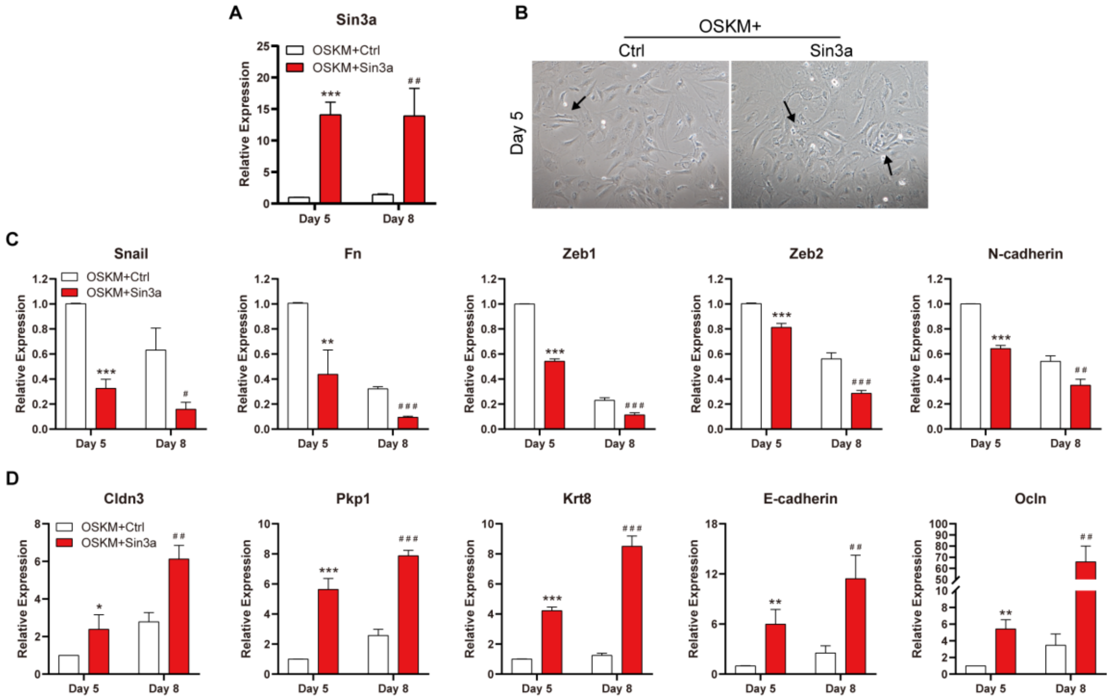

Figure S3. Sin3a overexpression facilitates MET process. Related to Figure 3.

(A) qRT–PCR analysis of Sin3a expression at Days 5 and 8 of reprogramming.

(B) Representative images of cell morphology at Day 5 of reprogramming. Black arrows indicate the representative cell-cell contact morphology.

(C, D) qRT–PCR analysis of the expression changes of mesenchymal markers (C) and epithelial markers (D) at Day 5 and Day 8 of reprogramming after Sin3a overexpression.

Significance was estimated by student's unpaired t-test (n=3). *P < 0.05, **P < 0.01, and ***P < 0.001 versus the control group at Day 5. $^{\#}$P < 0.05, $^{\#\#}$P < 0.01, and $^{\#\#\#}$P < 0.001 versus the control group at Day 8. Error bars represent SD.

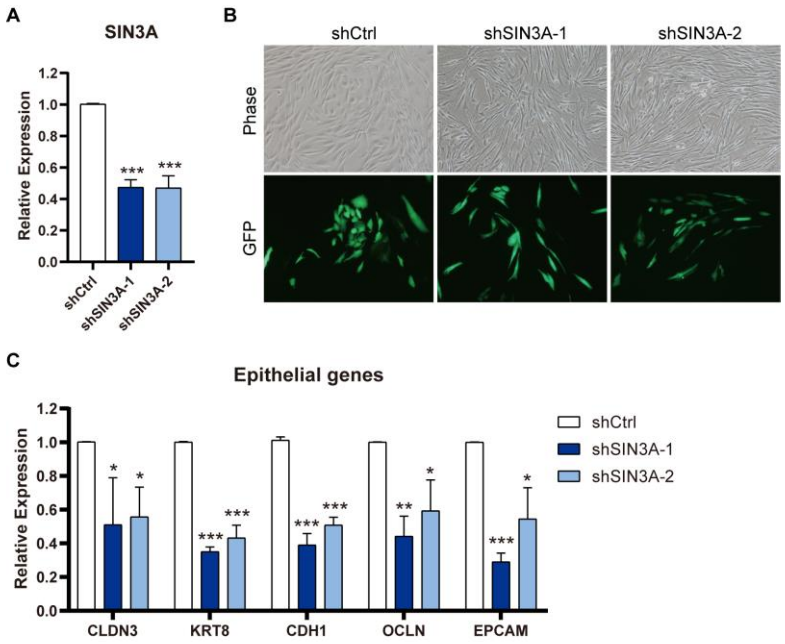

Figure S4. SIN3A knockdown hinders the acquisition of epithelial features during the reprogramming of human skin fibroblasts. Related to Figure 3.

(A) qRT–PCR analysis of SIN3A expression at Day 12 of reprogramming. Human skin fibroblasts were infected with scramble (shCtrl) or shSIN3A (shSIN3A-1 or shSIN3A-2) lentivirus.

(B) Representative images of the cell morphology of the control and SIN3A knockdown groups at Day 12. The GFP signal indicated the exogenous expression of reprogramming factors.

(C) qRT–PCR analysis of epithelial gene expression at Day 12 of human fibroblast reprogramming after SIN3A knockdown.

Significance was estimated by student's unpaired t-test (n=3). *P < 0.05, **P < 0.01, and ***P < 0.001 versus the control group. Error bars represent SD.

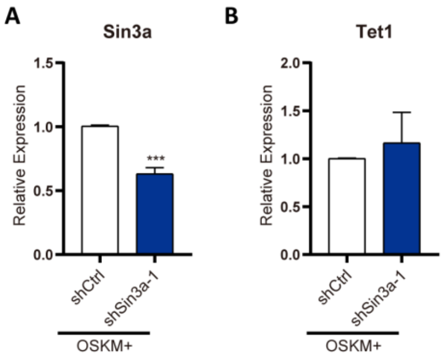

Figure S5. The expression levels of Sin3a and Tet1 upon Sin3a knockdown. Related to Figure 4.

(A and B) qRT–PCR analysis of the expression of Sin3a (A) and Tet1 (B) at Day 5 of reprogramming.

Significance was estimated by student's unpaired t-test (n=3). ***P < 0.001 versus the control group. Error bars represent SD.

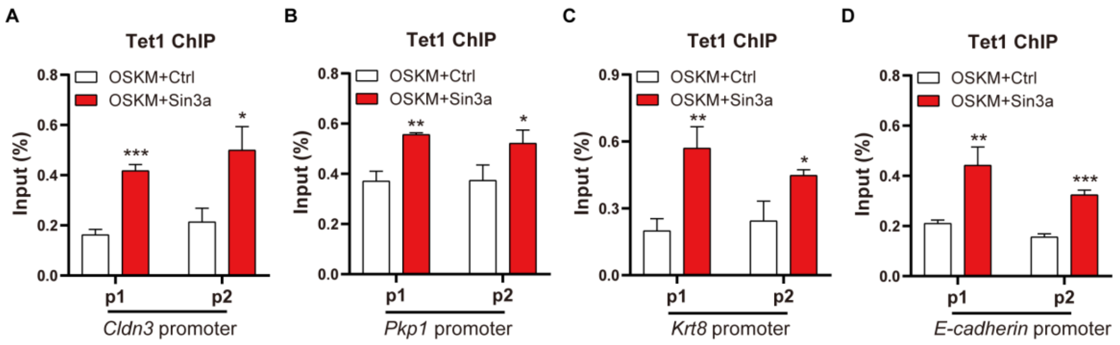

Figure S6. Sin3a overexpression increases Tet1 enrichment at the promoter of epithelial genes. Related to Figure 4.

(A-D) ChIP-qPCR analysis of Tet1 occupation at the promoter of epithelial markers (*Cldn3* (A), *Pkp1* (B), *Krt8* (C), and *E-cadherin* (D)) after Sin3a overexpression. The samples were normalized to input DNA.

Significance was estimated by student's unpaired t-test. Error bars represent SD (n=3). *P < 0.05, **P < 0.01, and ***P < 0.001 versus the control group.

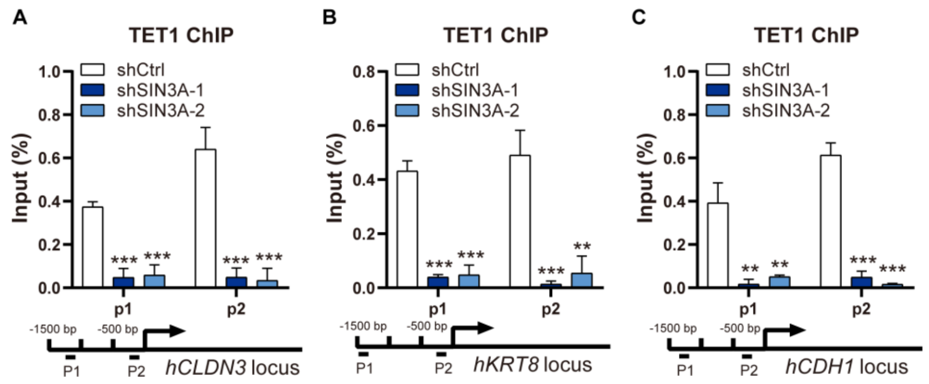

Figure S7. SIN3A knockdown leads to impaired TET1 occupation at the promoter of epithelial genes during the reprogramming of human skin fibroblasts. Related to Figure 4.

(A-C) ChIP-qPCR analysis of TET1 occupation at the promoter of epithelial markers (*CLDN3* (A), *KRT8* (B), and *CDH1* (C)) at Day 12 of human skin fibroblast reprogramming. The samples were normalized to input DNA.

Significance was estimated by student's unpaired t-test (n=3). **P < 0.01 and ***P < 0.001 versus the control group. Error bars represent SD.

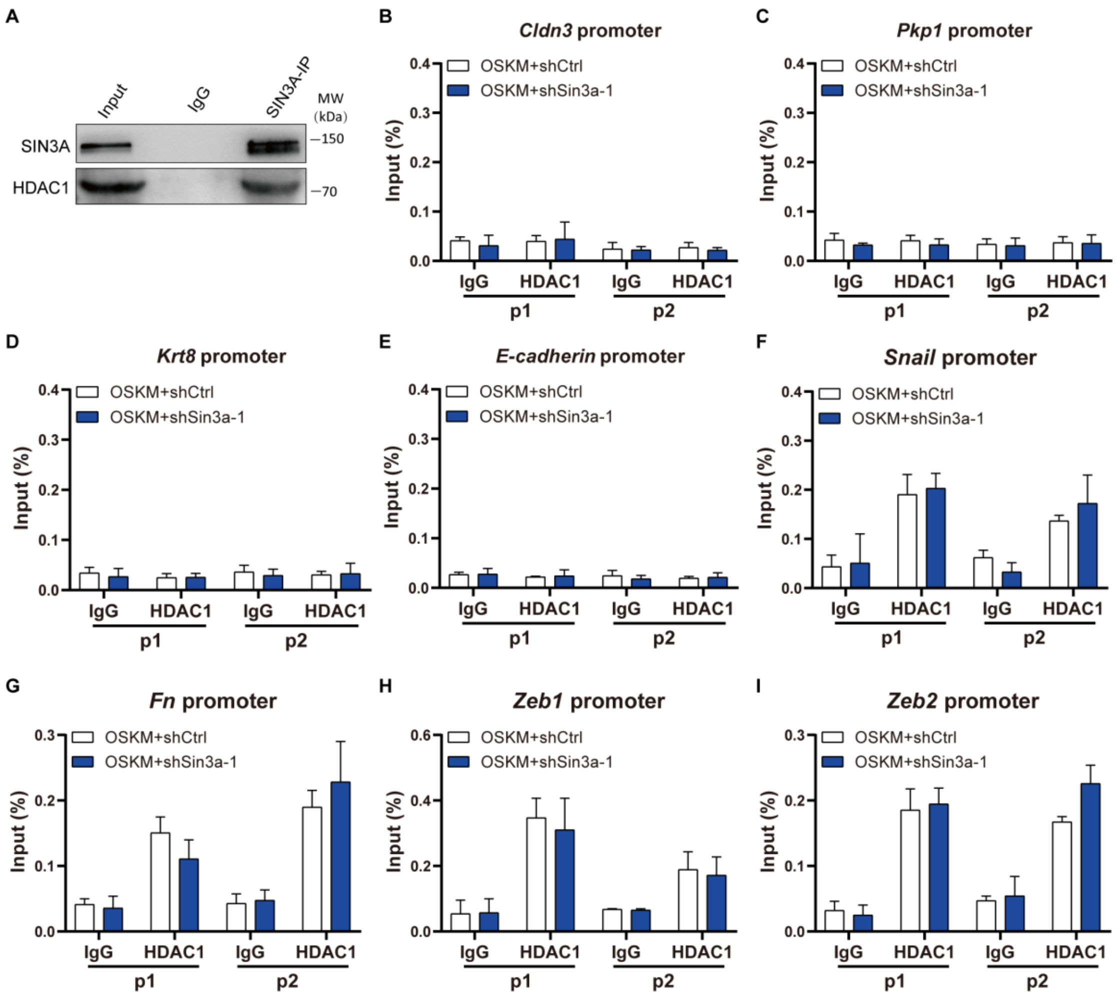

Figure S8. The enrichment of HDAC1 at the promoter of mesenchymal genes is independent of Sin3a. Related to Figure 4.

(A) Co-IP analysis of the interaction of endogenous SIN3A and HDAC1 at Day 5 of reprogramming. 10% of total lysates were used as input. Mouse IgG antibody was used as the negative control.

(B-E) ChIP-qPCR analysis (n=4) of HDAC1 enrichment at the promoter of epithelial markers (*Cldn3* (B), *Pkp1* (C), *Krt8* (D), and *E-cadherin* (E)) at Day 5 of reprogramming. The samples were normalized to input DNA. Mouse IgG antibody was used as negative control.

(F-I) ChIP-qPCR analysis (n=3) of HDAC1 occupation at the promoter of mesenchymal genes (*Snail* (F), *Fn* (G), *Zeb1* (H), and *Zeb2* (I)) at Day 5 of reprogramming.

Significance was estimated by student’s unpaired t-test. Error bars represent SD.

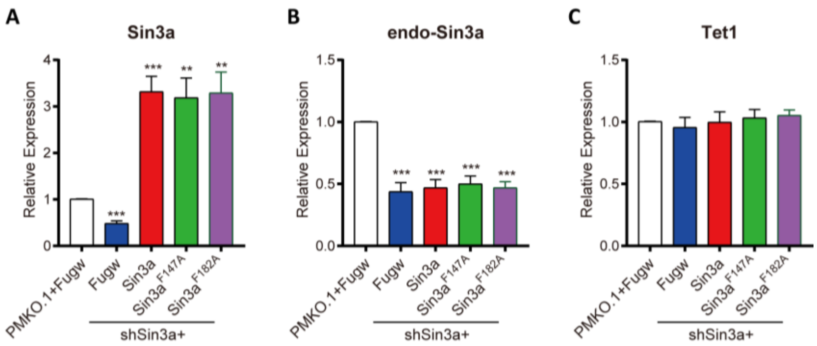

Figure S9. Expression analysis of Sin3a and Tet1 in reprogramming cells overexpressing Sin3a or mutants. Related to Figure 5.

(A) qRT–PCR analysis of Sin3a expression at Day 5 of reprogramming with wild-type Sin3a or Sin3a mutants (Sin3a^{F147A} and Sin3a^{F182A}) overexpression in shSin3a-1 cells.

(B and C) qRT–PCR analysis of the expression of endogenous Sin3a (B) and Tet1 (C) at Day 5 of reprogramming.

Significance was estimated by student's unpaired t-test. Error bars represent SD (n=3). **P < 0.01 and ***P < 0.001 versus PMKO.1+Fugw group.

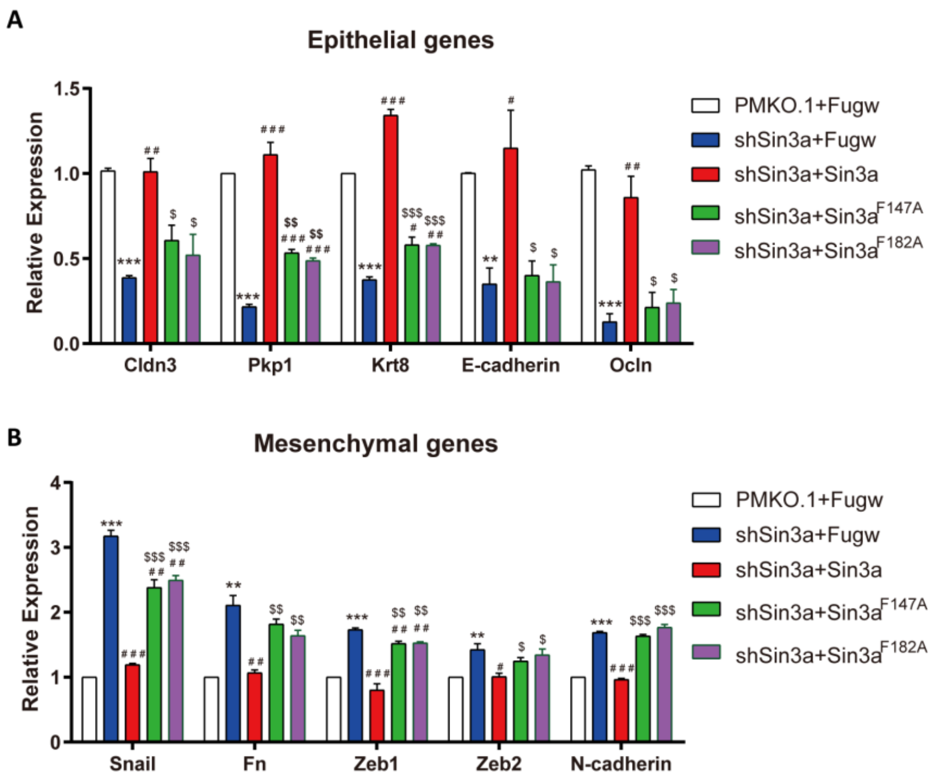

Figure S10. The rescue effect of epithelial and mesenchymal gene expression after overexpressing wild-type Sin3a or mutants. Related to Figure 6.

(A) qRT–PCR analysis of epithelial gene expression at Day 5 of reprogramming in shSin3a-1 cells overexpressing wild-type Sin3a or Sin3a mutants (Sin3a^{F147A} and Sin3a^{F182A}).

(B) qRT–PCR analysis of mesenchymal gene expression at Day 5 of reprogramming in shSin3a-1 cells overexpressing wild-type Sin3a or Sin3a mutants (Sin3a^{F147A} and Sin3a^{F182A}).

Significance was estimated by student's unpaired t-test. Error bars represent SD (n=3). **P < 0.01 and ***P < 0.001 versus PMKO.1+Fugw group (the white column), $^{\#}P < 0.05$, $^{\#\#}P < 0.01$, and $^{\#\#\#}P$ < 0.001 versus shSin3a+Fugw group (the blue column), $^{\$}P < 0.05$, $^{\$\$}P < 0.01$, and $^{\$\$\$}P < 0.001$ versus the shSin3a+Sin3a group (the red column).

Supplementary Tables

Table S1. Primers used for vector construction.

Gene symbols	Primer sequences	F/R
shCtrl	CCGGCCTAAGGTTAAGTCGCCCTCGCTCGAGCGAGGGCGACTTAACCTTAGGTTTTTG	F
shCtrl	AATTCAAAAACGAGGGCGACTTAACCTTAGGCTCGAGCCTAAGGTTAAGTCGCCCTCG	R
shSin3a-1	CCGGCTGCTGAGAAGGTTGATTCTGCTCGAGCAGAATCAACCTTCTCAGCAGTTTTTG	F
shSin3a-1	AATTCAAAAACTGCTGAGAAGGTTGATTCTGCTCGAGCAGAATCAACCTTCTCAGCAG	R
shSin3a-2	CCGGGCTGTTCCGATTGTCCTTAAACTCGAGTTTAAGGACAATCGGAACAGCTTTTTG	F
shSin3a-2	AATTCAAAAAGCTGTTCCGATTGTCCTTAAACTCGAGTTTAAGGACAATCGGAACAGC	R
shTet1-1	CCGGCAGGTGGGTTTGCAGAAACAACTCGAGTTGTTTCTGCAAACCCACCTGTTTTTG	F
shTet1-1	AATTCAAAAACAGGTGGGTTTGCAGAAACAACTCGAGTTGTTTCTGCAAACCCACCTG	R
shTet1-2	CCGGAAGGTTGGATTTGATCACACACTCGAGTGTGTGATCAAATCCAACCTTTTTTTG	F
shTet1-2	AATTCAAAAAAAGGTTGGATTTGATCACACACTCGAGTGTGTGATCAAATCCAACCTT	R
Flag-Sin3a	CCGACCGGTATGGATTACAAGGATGACGACGATAAGAAGCGACGTTTGGATGACCAGG	F
Flag-Sin3a	CCGGAATTCTTAAGGGGCTTTGAATACTGTGCCG	R
F147A 1F	CCGACCGGTATGGATTACAAGGATGACGACGATAAGAAGCGACGTTTGGATGACCAGG	F
F147A 1R	TTCCTTCATGATGTCAAGGGCATCATTGTAGACCTGAGGCTG	R
F147A 2F	CAGCCTCAGGTCTACAATGATGCCCTTGACATCATGAAGGAA	F
F147A 2R	CCGGAATTCTTAAGGGGCTTTGAATACTGTGCCG	R
F182A 1F	CCGACCGGTATGGATTACAAGGATGACGACGATAAGAAGCGACGTTTGGATGACCAGG	F
F182A 1R	AGGAGGCAAGAAGGTGTTGGCGCCCATGATCAGATCAGGGTG	R
F182A 2F	CACCCTGATCTGATCATGGGCGCCAACACCTTCTTGCCTCCT	F
F182A 2R	CCGGAATTCTTAAGGGGCTTTGAATACTGTGCCG	R

Table S2. Primers for qRT-PCR assays.

Gene symbols	Primer sequences	F/R
Sin3a	AGTGTCAACGTGGTCGAGAG	F
	ATGCAGACGCTTCTTGCTTAC	R
Endo-Sin3a	AGCCAGGATTTGATGAT	F
	AGAAGAGGGAAGAAGGTG	R
Tet1	ACACAGTGGTGCTAATGCAG	F
	AGCATGAACGGGAGAATCGG	R
Endo-Oct4	GCCTTTCCCTCTGTTCCC	F
	CCCTTGCCTTGGCTCAC	R
Endo-Sox2	TGTGAGGGCTGGACTGC	F
	TGGATGGGATTGGTGGT	R
Nanog	CAGGTGTTTGAGGGTAGCTC	F
	CGGTTCATCATGGTACAGTC	R
Rex1	TCCAAGGAGCTGAACTCCT	F
	CGTCTTGCTTTAGGGTCAGTT	R
Sall4	TGGTCCAGCCAATGACTCT TCCTT	F
	TCGGATAAATGTTGGAGGGAGGCT	R
Esrrb	CATGAAATGCCTCAAAGTGGG	F
	AAATCGGCAGGTTCAGGTAG	R
Tcl1	TGGGAGAAGCACGTGTACTTGGAT	F
	GTTGCCACATTAAAGGCAGCTCGT	R
Snail	CACACGCTGCCTTGTGTCT	F
	GGTCAGCAAAAGCACGGTT	R
Fn	ATGTGGACCCCTCCTGATAGT	F
	GCCCAGTGATTTCAGCAAAGG	R
Zeb1	GCTGGCAAGACAACGTGAAAG	F
	GCCTCAGGATAAATGACGGC	R
Zeb2	ATTGCACATCAGACTTTGAGGAA	F
	ATAATGGCCGTGTCGCTTCG	R
N-cadherin	AGCGCAGTCTTACCGAAGG	F
	TCGCTGCTTTCATACTGAACTTT	R
Cldn3	ACCAACTGCGTACAAGACGAG	F
	CAGAGCCGCCAACAGGAAA	R
Pkp1	AACCACTCTCCGCTCAAGAC	F
	CTTCTGCCGTTTGACGGTCAT	R
Krt8	TCCATCAGGGTGACTCAGAAA	F
	CCAGCTTCAAGGGGCTCAA	R
E-cadherin	CAGGTCTCCTCATGGCTTTGC	F
	CTTCCGAAAAGAAGGCTGTCC	R
Ocln	TTGAAAGTCCACCTCCTTACAGA	F

	CCGGATAAAAAGAGTACGCTGG	R
hCLDN3	CACCATTATCCGGGACTTCTAC	F
	GGTGGCCGTGTACTTCTTCTC	R
hKRT8	ACATGGACAACATGTTCGAGAG	F
	GGACAAATTCGTTCTCCATCTC	R
hCDH1	TAGTCTGAGCTCCCTGAACTCC	F
	GTACATGTCAGCCAGCTTCTTG	R
hOCLN	GATTCGGATCCTGTCTATGCTC	F
	TAGCTACCAAAGCCACTTCCTC	R
hEPCAM	ATTGTGGTTGTGGTGATAGCAG	F
	CACCCATCTCCTTTATCTCAGC	R

Table S3. Primers for ChIP-qPCR assays.

Gene symbols	Primer sequences	F/R
Cldn3 p1	CAAAGTGGCAAAGAGAACAGTG	F
	ACAGGGTTTCTCTGTTGTAGCC	R
Cldn3 p2	TGCCATCCATCTATTAGGGTTT	F
	CCTTTCTCCATGAGACACTCCT	R
Pkp1 p1	CTGTGACTGACGTCTGGAGAAC	F
	TATGCACCAAGGAGACTGACTG	R
Pkp1 p2	AGGGTGTGATGCCTTTAAGAAA	F
	CTCCCTACTATGTGCCAAGGAC	R
Krt8 p1	TTAATTGAGATCCACCCAATCC	F
	ACCTGTGAGAGGTTGGAGACTG	R
Krt8 p2	GGTCTGGGTAGGAAAGGAGAAT	F
	TGAGGAATGAATGAAACACAGG	R
E-cadherin p1	CAGTGCCATACAGGCTAGTGAC	F
	GTACCACCCTCAGCACAGTGTA	R
E-cadhein p2	TACCAGGGACAGAATTCTCAGG	F
	ATCGTGTAGCAAGTCAACGTGT	R
Snail p1	AGGTGTGGATCTGGAAGAGAAA	F
	TTTGACTTTTCTCCATCCCCTA	R
Snail p2	CACCTCCTTTCATCAAATCACA	F
	TACACAGCCCTGAACAAAACAC	R
Fn p1	GGTTCCCCTGATCCTTATTTTC	F
	CAAAGCACTGAGCCAACTAACA	R
Fn p2	AGACTTCTTGCACTGGCTTTTC	F
	AGACACGCAAAACCTCTCATTT	R
Zeb1 p1	TTAACATCTCATGGTTGGTGCT	F
	TTTTTATGGCGATACACACCAG	R
Zeb1 p2	CTATTCTCCGCTACTCCACCAC	F
	GAGGCTTTACGACATCACCTTC	R
Zeb2 p1	TTCTTTGTCCTGAAAGAATGAATC	F
	AGGCCAAGAAAAATAAAGATGC	R
Zeb2 p2	TGGACGTATAAAGTAGGCACCA	F
	CCCTTTATCCAGATTTTCTTGC	R
hCLDN3 p1	TTAGGTGGTTCCCCTCCTTTAT	F
	GTGTCAGTAAAGCCCAGGACTC	R
hCLDN3 p2	CTCATGCCTGCTTCCTAGATTG	F
	GGATGGAGCTTAGAGGGAAGAT	R
hKRT8 p1	GGCAGAAGGTTGGTATTGTTTC	F
	GGATTTGCACACTGTCACCTTA	R
hKRT8 p2	GATAATGCCCTCAAAAACTTGC	F

	AACCTTGCTTGTTGCCATAGAT	R
hCDH1 p1	ATGCCTGGCCCTATTGTTACTA	F
	CAGTTTCTCCACCCTCCTAATG	R
hCDH1 p2	AGCTTGGGTGAAAGAGTGAGAC	F
	GCTCACTAAGACCTGGGATCAG	R

Table S4. Primers for hMeDIP-qPCR assays.

Gene symbols	Primer sequences	F/R
Cldn3	CAAAGTGGCAAAGAGAACAGTG	F
	ACAGGGTTTCTCTGTTGTAGCC	R
Krt8	TTAATTGAGATCCACCCAATCC	F
	ACCTGTGAGAGGTTGGAGACTG	R
Pkp1	AGGGTGTGATGCCTTTAAGAAA	F
	CTCCCTACTATGTGCCAAGGAC	R
E-cadherin	TACCAGGGACAGAATTCTCAGG	F
	ATCGTGTAGCAAGTCAACGTGT	R
